# Impact of a Panel of 88 Single Nucleotide Polymorphisms on the Risk of Breast Cancer in High-Risk Women: Results From Two Randomized Tamoxifen Prevention Trials

**DOI:** 10.1200/JCO.2016.69.8944

**Published:** 2016-12-27

**Authors:** Jack Cuzick, Adam R. Brentnall, Corrinne Segal, Helen Byers, Caroline Reuter, Simone Detre, Elena Lopez-Knowles, Ivana Sestak, Anthony Howell, Trevor J. Powles, William G. Newman, Mitchell Dowsett

**Affiliations:** Jack Cuzick, Adam R. Brentnall, Caroline Reuter, and Ivana Sestak, Centre for Cancer Prevention, Wolfson Institute of Preventive Medicine, Queen Mary University of London; Corrinne Segal, The Institute of Cancer Research; Corrinne Segal, Simone Detre, Elena Lopez-Knowles, and Mitchell Dowsett, Royal Marsden Hospital; Trevor J. Powles, Cancer Centre London, London; Helen Byers and William G. Newman, University of Manchester and Central Manchester Foundation Trust; and Anthony Howell, The Christie NHS Foundation Trust, Manchester, United Kingdom.

## Abstract

**Purpose:**

At least 94 common single nucleotide polymorphisms (SNPs) are associated with breast cancer. The extent to which an SNP panel can refine risk in women who receive preventive therapy has not been directly assessed previously.

**Materials and Methods:**

A risk score on the basis of 88 SNPs (SNP88) was investigated in a nested case-control study of women enrolled in the International Breast Intervention Study (IBIS-I) or the Royal Marsden study. A total of 359 women who developed cancer were matched to 636 controls by age, trial, follow-up time, and treatment arm. Genotyping was done using the OncoArray. Conditional logistic regression and matched concordance indices (mC) were used to measure the performance of SNP88 alone and with other breast cancer risk factors assessed using the Tyrer-Cuzick (TC) model.

**Results:**

SNP88 was predictive of breast cancer risk overall (interquartile range odds ratio [IQ-OR], 1.37; 95% CI, 1.14 to 1.66; mC, 0.55), but mainly for estrogen receptor–positive disease (IQ-OR, 1.44; 95% CI, 1.16 to 1.79; *P* for heterogeneity = .10) versus estrogen receptor–negative disease. However, the observed risk of SNP88 was only 46% (95% CI, 19% to 74%) of expected. No significant interaction was observed with treatment arm (placebo IQ-OR, 1.46; 95% CI, 1.13 to 1.87; tamoxifen IQ-OR, 1.25; 95% CI, 0.96 to 1.64; *P* for heterogeneity = .5). The predictive power was similar to the TC model (IQ-OR, 1.45; 95% CI, 1.21 to 1.73; mC, 0.55), but SNP88 was independent of TC (Spearman rank-order correlation, 0.012; *P* = .7), and when combined multiplicatively, a substantial improvement was seen (IQ-OR, 1.64; 95% CI, 1.36 to 1.97; mC, 0.60).

**Conclusion:**

A polygenic risk score may be used to refine risk from the TC or similar models in women who are at an elevated risk of breast cancer and considering preventive therapy. Recalibration may be necessary for accurate risk assessment.

## INTRODUCTION

Breast cancer is associated with mutations in rare but highly penetrant dominant genes such as *BRCA1* and *BRCA2* and, to a lesser extent, genes such as *CHEK2* and *PALB2*.^1^ Although important for families with a history of the disease, they are too rare to be of much use for risk stratification in the general population. At least 94 common breast risk single nucleotide polymorphisms (SNPs) have been shown to be associated with breast cancer.^2-18^ It is anticipated that many more will be found in future,^1^ but they are expected to be individually much less predictive. Each SNP found to date only confers a small relative risk, but together, they can usefully stratify risk in the general population.^19^

Accurate risk assessment is important for many decisions about preventive interventions, such as increased screening and preventive therapy.^20^ Risk evaluators available include the Breast Cancer Risk Assessment Tool (Gail model), Tyrer-Cuzick (TC), Breast Cancer Surveillance Consortium, and the Breast and Ovarian Analysis of Disease Incidence and Carrier Estimation Algorithm models.^21-24^ SNP panels are most likely to be of use in clinical practice if they can be combined with such models; therefore, it is important to measure how much additional information they add.

Previous work in this area has been done using case-control studies of mainly postmenopausal women. Wacholder et al^25^ showed that fitting a SNP10 risk score in addition to Gail model factors yielded a modest improvement. Mealiffe et al^26^ found a similar result in in the Women’s Health Initiative trial using a predefined SNP7 panel, and Lee et al^27^ reported that a SNP51 panel added to Gail model risk factors in Asian women. Vachon et al^28^ showed that a predefined SNP76 panel added information to the Breast Cancer Surveillance Consortium model in three case-control studies. Mavaddat et al^29^ fitted a SNP77 panel in a large case-control analysis of women from the general population. Dite et al^30^ reported that a predefined SNP77 panel was informative for women at increased risk for breast cancer as a result of classic risk factors from the Australian Breast Cancer Family Registry.

Our focus is the extent to which an SNP panel can refine risk in women already at an increased risk for breast cancer as a result of classic factors and who may receive preventive therapy and to assess whether the preventive effect of tamoxifen varied according to SNP-derived estimates of risk. A previous study of the first of these issues has been conducted by Vachon et al.^31^ They used a nested case-control study within the National Surgical Adjuvant Breast and Bowel Project P-1 and P-2 prevention trials, on the basis of women receiving tamoxifen or raloxifene (no placebo), and found that a 75-SNP panel had good univariable power for predicting subsequent risk and added accuracy to the Gail model. However, they did not compare directly with women who did not receive treatment and therefore were unable to directly test for a difference between women receiving treatment or not, nor could they estimate the relative information from the SNP panel compared with classic factors.

A consortium has designed a custom Illumina (San Diego, CA) array (OncoArray), with more than half a million SNPs, including most known breast-cancer risk SNPs. In this article, we present the results from an SNP score on the basis of this assay, using a nested case-control study of women recruited into the International Breast Intervention Study (IBIS-I)^32^ and Royal Marsden^33^ randomized tamoxifen prevention trials. The SNPs used were those for which the breast cancer risk has been previously validated and are directly available or have close surrogates on the OncoArray. The main objectives were to evaluate the performance of the SNP panel using a population at increased risk as a result of classic risk factors (including a family history or prior proliferative benign tissue diagnosis); compare and combine this with risk estimates from the TC model,^22^ which uses classic phenotypic factors; and to conduct subgroup analyses to assess the performance by treatment allocation (tamoxifen or placebo) and estrogen-receptor (ER) status of the tumors that subsequently occurred.

## MATERIALS AND METHODS

### Patients

Women were recruited into the IBIS-I and Royal Marsden (Marsden) trials, as previously described^32,33^ (Data Supplement). Both trials were double blind with women randomly assigned to receive tamoxifen (20 mg/day) or placebo for 5 years (IBIS-I) or 5 to 8 years (Marsden). Subsequent cancers were ascertained by clinic visits during the treatment period and thereafter by questionnaire, cancer registry follow-up, and/or clinic visits. All women provided informed consent, including for use of their samples in future research. The trials are registered at www.controlled-trials.com as ISRCTN91879928 (IBIS-I) and ISRCTN07027313 (Marsden).

### Specimen Characteristics

Blood samples were taken at baseline from all women in IBIS-I and were stored at −70°C. The baseline blood samples for Marsden were destroyed by a fire, and subsequent blood samples were collected where possible and stored at −70°C. However, they were not available for 38 cases, where tissue samples from formalin-fixed paraffin-embedded blocks were used. They were included only in a sensitivity analysis.

### Assay Methods

We used 10 μL of extracted DNA from IBIS-I (concentration 100 ng/μL) and Marsden (50 ng/μL) samples on thirteen 96-well plates, including a separate plate for the formalin-fixed paraffin-embedded block samples. Two samples with known genotypes were used as internal controls for each plate. Assays were carried out blindly at Genome Quebec (Montreal, Canada), which was clinical service provider–certified by Illumina; the Illumina OncoArray was used, and the Illumina HTS (high-throughput sequencing) protocol was rigorously followed.

### Study Design

The primary end point was diagnosis of invasive breast cancer or ductal carcinoma in situ. The study was designed to use all cases from the trials with material available for genotyping and had more than 99% power to detect the expected polygenic score at a 5% level.^34^ The Marsden trial was a pilot for the IBIS-I trial, and the cohorts had similar characteristics. They were combined to increase precision and to provide greater power for subgroup analyses, as in previous analyses.^35^ Case-control matching was by study, age at baseline (± 2 years), treatment arm, and follow-up time, with two controls per case in IBIS-I and one in Marsden. IBIS-I recruited from 1992 to 2001, and Marsden recruited from 1986 to 1996. The end of follow-up for the current analysis was 2014 and 2010, respectively; median follow-up was 16.5 years for IBIS-I and 18.4 years for Marsden.

A polygenic score was used to provide an overall risk estimate.^26^ Three SNPs were excluded because they had no surrogate SNP on the OncoArray (*r*^2^ < .8 from the 1000 Genomes Project Central European panel^36^) and three SNPs failed quality control. We calculated the normalized odds ratio for each of the three SNP genotypes (no risk alleles, 1 risk allele, and 2 risk alleles) from published per-allele odds ratios, assuming independence and normalizing by an assumed risk allele frequency so that the average risk was unity.^16-18^ An overall SNP risk score for each woman (SNP88) was formed by multiplying the odds ratios for each of her 88 genotypes together (Data Supplement). The odds ratios for the TC risk^22^ (v7.02) at entry to each study were obtained by dividing the 10-year predicted odds for a person by that from the average population of a woman of the same age (Data Supplement).

### Analysis Methods

SNPs that failed in more than 2% of the samples were excluded, as well as samples that failed for more than 10 SNPs. Hardy-Weinberg equilibrium for each SNP in cases and controls was tested by assessing the observed number of homozygotes against expected using a binomial distribution. Sporadic assay failures in SNP88 were assigned the population risk of 1.0. Conditional logistic regression was used for the logarithm of the SNP88 score and TC risk, alone and in combination, assuming independence. A bivariable model was then used to assess the added value of the SNP88 score to the TC risk model. Interaction tests were used to examine subgroups. SNP88 and TC calibration was assessed primarily by regressing observed risks on expected risks. Calibration of SNP88 was further explored by deciles of the SNP88 in controls and for TC by the ratio of observed to expected cancers diagnosed in the entire placebo arm of the IBIS-I trial, overall and by decile (TC in the complete Marsden cohort was unavailable). Concordance indices for matched sets were used as a secondary measure of discrimination.^34^ The distribution of absolute 10-year risk was estimated by weighting cases and controls to reflect a 6% 10-year risk for the whole trial.^32,33^ An 8% 10-year risk cut point was chosen because this is the threshold recommended in the United Kingdom for offering preventive therapy.^20^ The reclassification of cases and controls about this cut point from TC was assessed using an SNP score that was recalibrated to the data. A sensitivity analysis was undertaken to impute risk from failed assays in the 88 SNPs by applying the Beagle algorithm (Data Supplement).^37^ A sensitivity analysis considered when tissue samples that did not fail more than 10 SNPs were included.

## RESULTS

**Fig 1.**
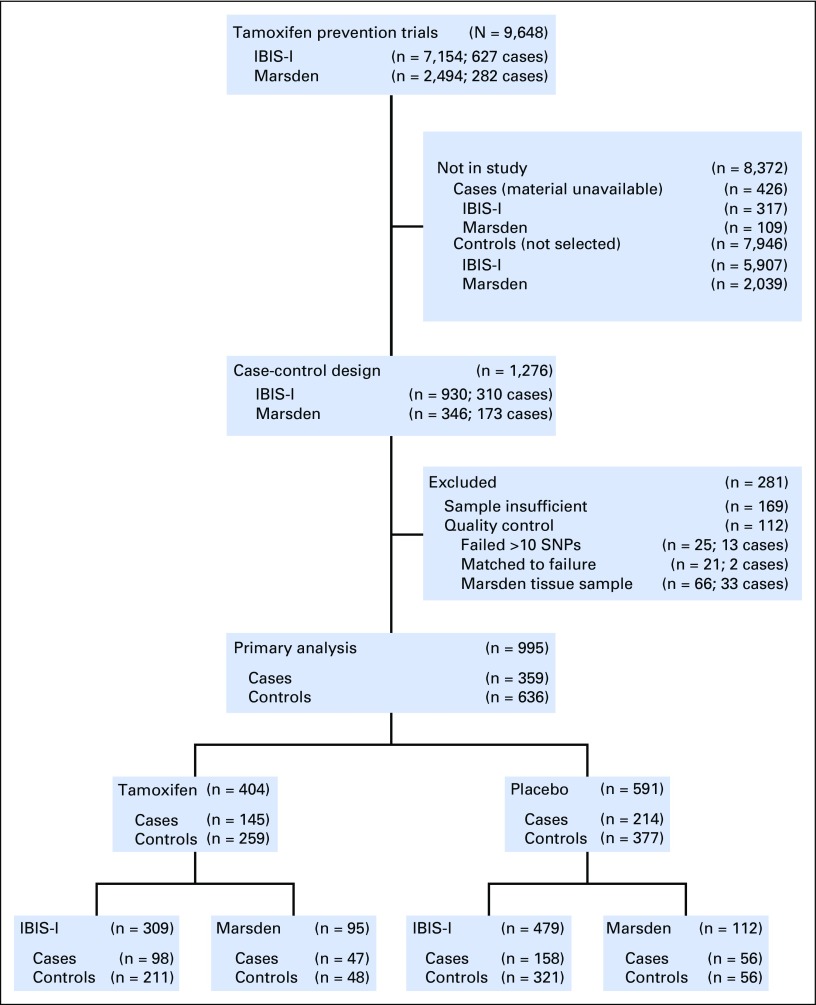
CONSORT diagram. IBIS-I, International Breast Intervention Study; Marsden, Royal Marsden study; SNPs, single nucleotide polymorphisms.

A CONSORT diagram of the source of patients is shown in Figure 1. A total of 1,276 women (483 cases, 793 controls) from both trials were initially selected for the case-control study. However, insufficient DNA was available in 169 samples, and the initial design was adjusted to ensure that all cases had matched controls. Twenty-five samples (2.3%) with more than 10 failures were excluded (Data Supplement), in addition to all tissue sample results, because tissue samples were more likely to have more than 10 SNP failures. This led to a primary analysis sample of 995 women (359 cases, 636 controls).

The sample characteristics of participants are listed in Table 1. Briefly, 41% were randomly assigned to tamoxifen and 59% to placebo; the imbalance reflects the lower proportion of cancers in women treated with tamoxifen. The majority of cancers were ER-positive (74%). At trial entry the median age was 50 years, 46% of participants were postmenopausal, and the median body mass index was 25.5 kg/m^2^. The characteristics in the two trials were broadly similar (Data Supplement).

**Table 1.**
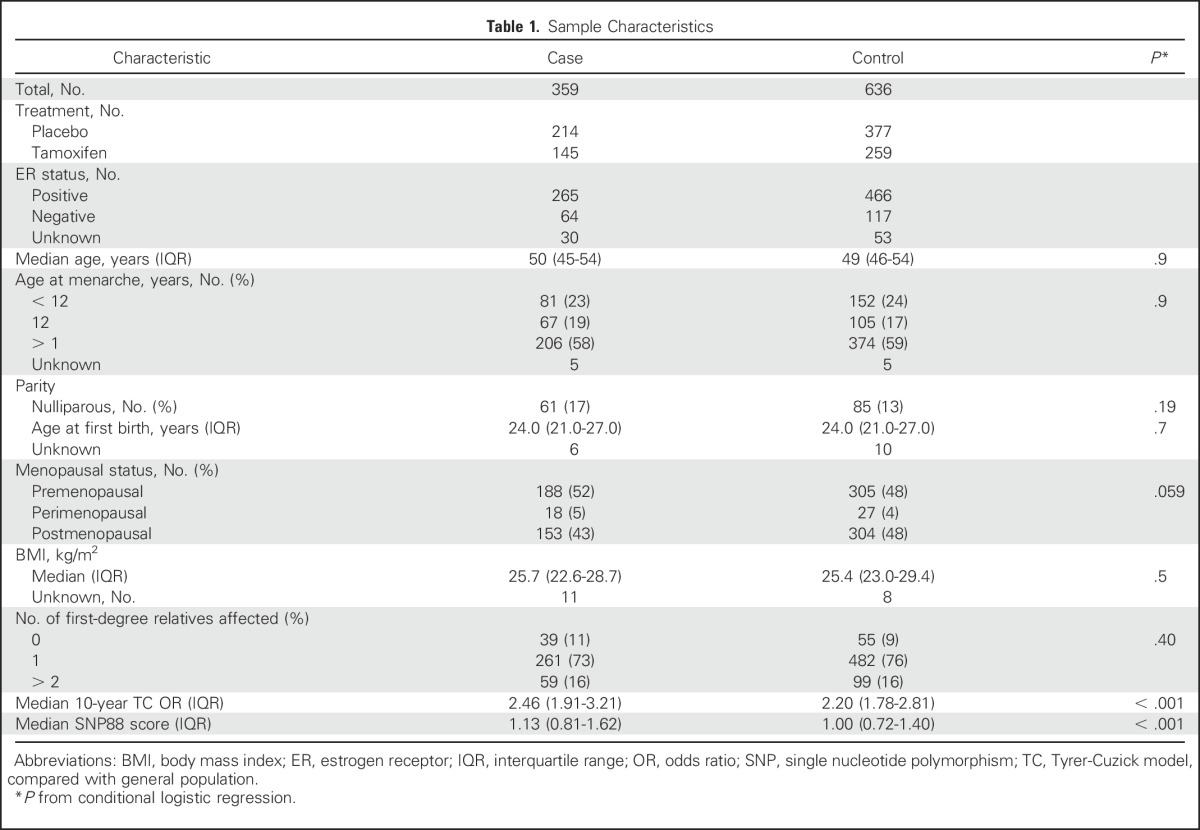
Sample Characteristics

Quality control of individual SNPs was assessed before calculating an SNP score. One SNP failed all samples (rs6678914), but the percentage of failed samples was less than 2% for all but two of the others, rs4808801 and rs2236007 (Data Supplement), which were excluded, leaving 88 SNPs for our primary analysis. The distribution of homozygotes satisfied the Hardy-Weinberg equilibrium in controls (*P* > .067 for each SNP; Data Supplement), and overall, the observed and expected number of homozygotes in controls were 36,177 and 36,176, respectively (*P* = 1.0). For cases, it was 20,349 and 20,443, respectively (*P* = .28).

As expected, the SNPs were mostly uncorrelated. However, three pairs had *r*^2^ > .1: rs554219 and rs7591516 (0.36); rs2363956 and rs8170 (0.25); and rs12662670 and rs2046210 (0.15). These are in line with expectations from the 1000 Genomes Project Central European panel. We made no adjustment for these small correlations in the SNP score.

SNP88 was predictive in the overall sample (interquartile range odds ratio [IQ-OR], 1.37; 95% CI, 1.14 to 1.66; matched concordance index, 0.55; *P* < .001), mainly for ER-positive disease (IQ-OR, 1.44; 95% CI, 1.16 to 1.79) versus ER-negative disease (IQ-OR, 0.99; 95% CI, 0.61 to 1.61; *P* = .10). No significant interaction was observed by treatment arm, but there were slightly stronger effects in untreated women (IQ-OR, 1.46; 95% CI, 1.13 to 1.87) compared with those receiving tamoxifen (IQ-OR, 1.25; 95% CI, 0.96 to 1.64; *P* = .5). The SNP score was nonsignificantly more predictive in IBIS-I than Marsden (*P* = .21; Table 2; Data Supplement), and so was TC (*P* = .6).

**Table 2.**
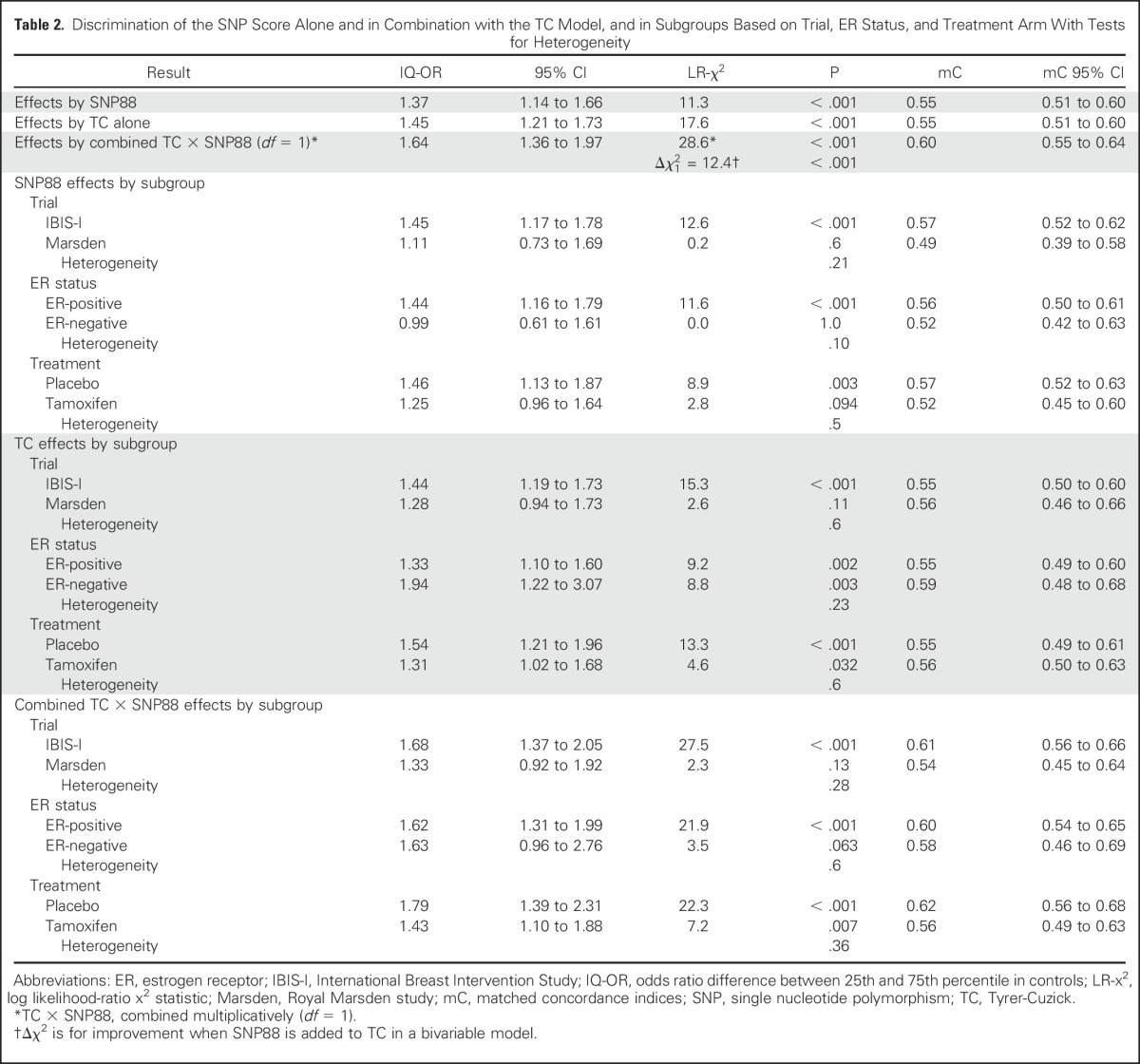
Discrimination of the SNP Score Alone and in Combination with the TC Model, and in Subgroups Based on Trial, ER Status, and Treatment Arm With Tests for Heterogeneity

**Fig 2.**
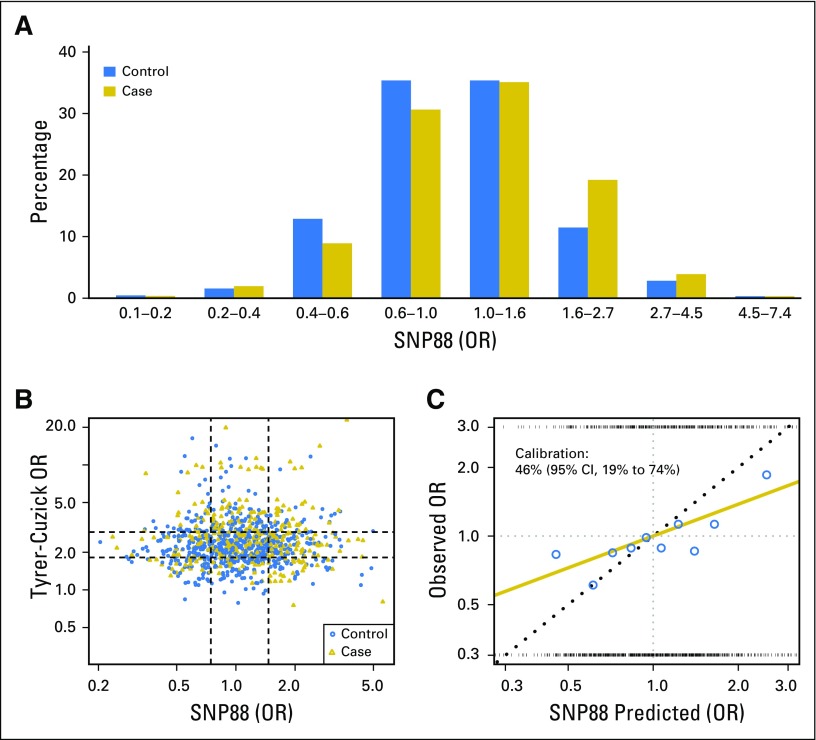
(A) Distribution of 88 single nucleotide polymorphisms risk score (SNP88) in cases and controls. (B) SNP88 and Tyrer-Cuzick odds ratios (ORs) for cases and controls; Spearman coefficient 0.012, *P* = .7, where each interquartile range in controls is represented by the dashed lines. (C) logistic regression fit (gold line) of observed versus predicted ORs from the SNP score in both arms, blue points are estimates from each decile of SNP88 in controls, and the rug plot at the top and bottom gives the observed SNP88 in cases and controls, respectively.

There was almost no correlation between the TC model and the SNP score (Spearman coefficient, 0.012; *P* = .7; Fig 2). SNP88 added significant independent information to TC (*P* < .001), and when combined multiplicatively, a substantial increase was seen in the predictive power (Table 2). The matched concordance index increased from 0.55 to 0.60, and the fit ($\chi_{1}^{2}$ = 28.6) was close to that for a full bivariable model ($\chi_{2}^{2}$ = 30.0). These results suggest that TC and SNP88 are largely independent and may be combined multiplicatively.

Calibration of the SNP score was assessed by comparing the model calculated odds ratios with those predicted by the SNP88 score in population deciles (Fig 2C). The observed SNP score risk was, in general, closer to unity than expected, and the log-odds ratio for SNP88 was estimated to be 46% (95% CI, 19% to 74%) of expected (Data Supplement). Calibration of the SNP score was slightly better for ER-positive cancers, where risk was 56% (95% CI, 23% to 88%) of expected. After allowance for TC in a bivariable model, the observed SNP88 risk was 49% (95% CI, 21% to 77%) of expected.

The TC model risk distribution was not significantly different from observed in this case-control study (slope, 71%; 95% CI, 37% to 106%; Data Supplement). In addition, TC was well calibrated for absolute risk in the IBIS-I placebo arm, where 363 cancers were diagnosed compared with 361 expected, and calibration was maintained by decile (Hosmer-Lemeshow $\chi_{8}^{2}$ = 13.7; *P* = .09; Data Supplement).

When reweighted to the original population, the percentage of women with 10-year predicted risk above 8% if untreated was 18% for TC and increased to 21% if recalibrated SNP88 was added. Table 3 lists the number of cases and controls who were reclassified when recalibrated SNP88 was added to TC. It shows that although the number in the ≥ 8% group was not substantially increased, those who were upgraded were at a significantly higher risk than were those who were downgraded (OR, 2.67; 95% CI, 1.12 to 6.60; *P* = .015).

**Table 3.**
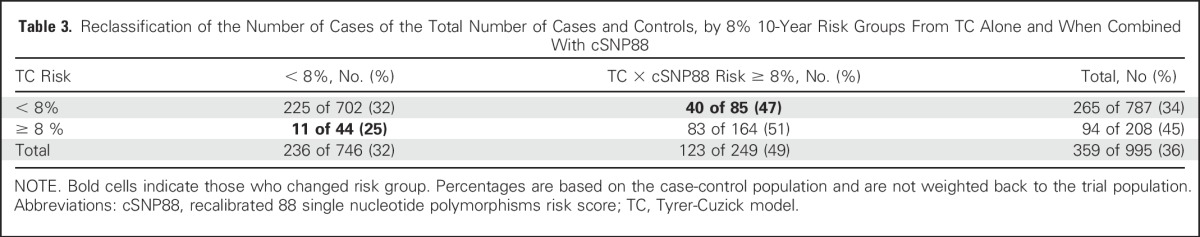
Reclassification of the Number of Cases of the Total Number of Cases and Controls, by 8% 10-Year Risk Groups From TC Alone and When Combined With cSNP88

No significant interactions between SNP88 and any subgroups were observed (all *P* > 0.1; Data Supplement). In sensitivity analyses, there was little difference when missing data were imputed (Data Supplement) or when the analysis included all tissue samples that met the quality control thresholds (Data Supplement).

## DISCUSSION

A polygenic SNP score on the basis of common low penetrance genetic polymorphisms provides additional risk information for women who are already at an increased risk for the disease as a result of classic factors. SNP88 was independent of the TC risk model, and no loss of relative predictive power was seen when it was used in combination with TC. However, the SNP panel seems to be in need of recalibration, at least when applied to high-risk women in similar populations. SNP88 had less than half the expected risk on the basis of multiplying published risks for individual SNPs. In addition, the spread of risks associated with SNP88 were of a smaller magnitude than those of TC (Table 1), especially after recalibration. Thus, although the SNP score had minimal ability to increase the number of classically high-risk women to be at more than 8% 10-year risk (Table 3), it did more accurately populate this group. The SNP score also did not predict which women would differentially benefit from tamoxifen.

One possible reason for the poor calibration of the SNP score is overfitting. Using the published odds ratios and CIs, we applied a James-Stein shrinkage estimator and estimated that this might account for overfitting of approximately 5% in calibration (data not shown). Thus, it is unlikely to explain most of the difference between observed and expected risks.

In general populations, polygenic scores have demonstrated good calibration. For example, Vachon et al^28^ used a SNP76 panel in a general population and found an OR per standard deviation (SD) of approximately 1.5, compared with 1.6 expected (calculated by computer simulation following Brentnall et al^19^): calibration was approximately 86% [log_e_(1.5)/log_e_(1.6)]. Dite et al^30^ reported a SNP77 panel in women with a family history. The OR per SD was approximately 1.4, which was only approximately 70% of expected. In the context of prevention trials, Vachon et al^31^ reported an odds ratio of approximately 1.8 for the difference between the top and bottom quintile of their SNP75 panel, compared with approximately 3.8 if the score had been well calibrated. Thus, overall, our results are not inconsistent with previous findings for women with an elevated risk of cancer, and it seems that recalibration of polygenic risk scores is needed for these women. A strong family history might change the relationship between SNPs and risk because risks for each SNP are different for *BRCA1* and *BRCA2* mutation carriers than for the general population.^38,39^ The percentage of *BRCA1* and *BRCA2* carriers was estimated to be 2.8% in Marsden and 1.9% in IBIS-I from the TC model; therefore, this is only a partial explanation.

A limitation of this study is that we could not assess the performance of SNPs in conjunction with mammographic breast density, which is another important component of risk.^40^ Also, the findings are only applicable to similar high-risk groups of women of the same age. It is likely that these findings apply to other risk models, such as the Gail model, because SNP scores are largely independent of the factors used.^25^

The study has several strengths. First, the cancers were diagnosed prospectively from an extended follow-up period. Second, we were able to compare the performance within the tamoxifen and placebo arms directly, without indirect comparisons with other studies. Third, we were able to assess how much an SNP score adds compared with a risk model on the basis of classic factors (except for age). This was not possible in the analysis from the P1 and P2 trials,^31^ because they matched on risk from classic factors. Another advantage of the current study is that the SNPs were genotyped directly (36 of 75 were imputed in Vachon et al^31^), so that a poor imputation procedure is not a possible reason for the calibration issue.

In conclusion, our findings extend earlier work to high-risk populations and indicate that SNP scores increase the accuracy of risk assessment for these women, but substantial recalibration seems to be required for accurate risk assessment.
